# Moderating effect of age on the relationship between physical health loss and emotional distress post-acute care in Japanese older hospitalized patients

**DOI:** 10.1186/s12877-024-04814-8

**Published:** 2024-03-02

**Authors:** Mio Shinozaki, Yasuyuki Gondo, Shosuke Satake, Masanori Tanimoto, Akiko Yamaoka, Marie Takemura, Izumi Kondo, Yutaka Arahata

**Affiliations:** 1 Department of Neurology, National Center for Geriatrics and Gerontology, 7-430 Morioka-Cho, Obu-City, Aichi 474-8511 Japan; 2 Graduate School of Human Science, Osaka University, Osaka, Japan; 3 Department of Frailty Research, National Center for Geriatrics and Gerontology, Aichi, Japan; 4 Department of Geriatric Medicine, National Center for Geriatrics and Gerontology, Aichi, Japan; 5 Department of Rehabilitation Medicine, National Center for Geriatrics and Gerontology, Aichi, Japan; 6 Center for Frailty and Locomotive Syndrome, National Center for Geriatrics and Gerontology, Aichi, Japan; 7 Assistive Robot Center, National Center for Geriatrics and Gerontology, Aichi, Japan

**Keywords:** Post-acute care, Emotional distress, Psychological adjustment, Physical illness and disability, Moderating effect, Very old age, Lifespan developmental changes

## Abstract

**Background:**

At present, there are no consistent findings regarding the association between physical health loss and mental health in older adults. Some studies have shown that physical health loss is a risk factor for worsening of mental health. Other studies revealed that declining physical health does not worsen mental health. This study aimed to clarify whether the relationship between physical health loss and emotional distress varies with age in older inpatients post receiving acute care.

**Methods:**

Data for this study were collected from 590 hospitalized patients aged ≥ 65 years immediately after their transfer from an acute care ward to a community-based integrated care ward. Emotional distress, post-acute care physical function, and cognitive function were assessed using established questionnaires and observations, whereas preadmission physical function was assessed by the family members of the patients. After conducting a one-way analysis of variance (ANOVA) and correlation analysis by age group for the main variables, a hierarchical multiple regression analysis was conducted with emotional distress as the dependent variable, physical function as the independent variable, age as the moderator variable, and cognitive and preadmission physical function as control variables.

**Results:**

The mean GDS-15 score was found to be 6.7 ± 3.8. Emotional distress showed a significant negative correlation with physical function in younger age groups (65-79 and 80-84 years); however, no such association was found in older age groups (85-89, and ≥ 90 years). Age moderated the association between physical function and emotional distress. Poor physical function was associated with higher emotional distress in the younger patients; however, no such association was observed in the older patients.

**Conclusions:**

Age has a moderating effect on the relationship between physical health loss and increased emotional distress in older inpatients after acute care. It was suggested that even with the same degree of physical health loss, mental damage differed depending on age, with older patients experiencing less damage.

## Background

Generally, the risks of illness and disability increase with age. Illness and physical disability are major risk factors for depression [1–3]; and in many cases, older adults are reported to be emotionally stable [4–8] and are no more likely to become depressed than younger or middle-aged adults [9–12]. However, the relationship between physical and mental health loss in older patients after acute treatment remains unclear.

Why are older adults able to maintain their psychological health despite an increased risk of physical health loss? The two main explanations for this are discussed in this section. The first explanation is that the psychological impact of illness and disability as well as their meaning in life varies by life stage, and aging may be advantageous for maintaining mental health in the face of physical health loss [12–14]. It has been suggested that physical health loss in old age is predictable [14] and may be relatively normal and less damaging to life goals and satisfaction than in younger adults [12]. It has also been suggested that the ability to cope with illnesses may improve with age [14]. Compared to younger and middle-aged people, older adults are less likely to experience a substantial increase in depressive symptoms in the presence of diseases (such as cancer, stroke, heart disease, diabetes, and arthritis) or disabilities [12, 14].

Another explanation is that older adults have superior emotion regulation, and consequently, psychological functions related to emotions are less likely to decline in old age [6, 8, 15–17]. In fact, older adults’ evaluations of their own health are considerably more positive than the actual state, and this tendency is considered evidence that older adults can actively engage in cognitive emotion regulation (such as social comparison and goal disengagement) to prevent self-esteem decline and emotional deterioration when faced with declining physical health [18–21].

However, studies on frail older adults have indicated a high frequency of depression and anxiety. For example, 35% of hospitalized older patients and 12.4%–42% of institutionalized older adults have been reported to exhibit depressive symptoms [9, 10, 22, 23]. Furthermore, it has been reported that in the last few years of life, when frailty rapidly progresses, life satisfaction and happiness show a terminal decline while negative emotions increase in older adults [7, 24–28]. In other words, only older people with high levels of health can regulate their emotions and maintain good mental health, whereas frail and very old people with significantly deteriorating health status may have difficulty in regulating their emotions (and thereby suffer from worsening of their mental health). As far as the explanations for the psychological adjustment difficulties of older adults in the last few years of life are concerned, it has been suggested that psychological health may deteriorate due to overall reduced resources and increased vulnerability [7, 24, 25, 28–31]. Conversely, some researchers have argued that even the oldest individuals and centenarians who suffer from advanced frailty and are close to death have high psychological well-being and maintain psychological functioning related to emotional regulation and psychological adjustment [32, 33]. It is unclear whether older age is associated with less emotional distress when faced with physical health loss following acute treatment, or whether this age-related advantage disappears in advanced old age with advanced frailty.

The health status of older adults has improved over the past few decades in developed countries. However, many diseases that seriously affect life and life functions (such as stroke, cancer, dementia, and heart disease) still have a high risk of developing in old age [34–36], and those who suffer from such diseases may lose health even at an early age and die before reaching full life expectancy. As noted above, the ability to lose physical health is important for subsequent psychological adjustment [14]. Therefore, facing health loss due to illness or disability in their 60s and 70s during the first half of their life may cause psychological distress and maladjustment similar to that in younger adults. However, previous studies have not sufficiently clarified whether there is a difference in emotional distress related to health losses between the early and later stages of old age after acute treatment.

This study aimed to clarify whether the relationship between loss of physical health and emotional distress varies with age. To this end, we examined the association between physical functioning and emotional distress by age group and the moderating effect of age on the relationship between physical functioning and emotional distress in a Japanese sample of older inpatients immediately after they received acute care.

If the psychological damage due to illness and disability varies with age, as noted in previous studies [12–14], we hypothesized that emotional distress would be lower in older patients than in younger patients, even with similar levels of health loss.

## Methods

### Participants

Between July 2015 and September 2020, participants were recruited among hospitalized patients aged 65 years or older immediately after being transferred from an acute care unit to a community-based integrated care ward at the National Center for Geriatrics and Gerontology.

In this institution, patients who were not discharged immediately after acute care because of the need to coordinate the care services they would receive after discharge or because of the need for rehabilitation or improved nutrition, patients with advanced frailty or comorbidities (e.g., diabetes and bone fractures), patients over 80 years of age, and those with cognitive decline were transferred to the community-based integrated care ward for continuous care until discharge.

The study was explained, and consent was obtained at the time of transfer to the community care unit. Patients with terminal illnesses who were predicted to die within three months and those with conditions such as severe respiratory distress, high-level pain, and decreased level of consciousness during the aforementioned transfer to the community-based integrated care ward were not included in the participant list. Patients who were already taking antidepressants or mood stabilizers at the time of transfer to the community care unit were also excluded because of the potential impact of these medications on the GDS-15 scores. Patients who were taking antidepressants or mood stabilizers in the past but were not taking them at the time of transfer to the ward, or patients who had symptoms of depression but were not taking antidepressants or mood stabilizers were included in this study.

**Fig. 1:**
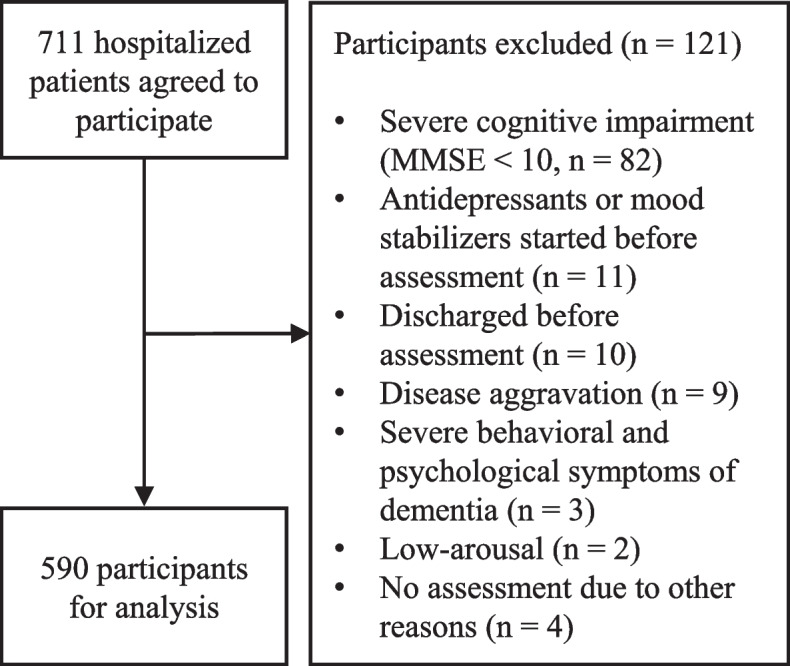
Flowchart of selection process of analysis participants

In total, 711 patients agreed to participate in the study. Since it has been pointed out that the 15-item version of the Geriatric Depression Scale (GDS-15) is useful for assessing depression in older adults with Mini-Mental State Examination (MMSE) scores of 10 or higher [37], 82 patients with MMSE scores of 9 or lower were excluded. Eleven patients in whom antidepressants or mood stabilizers were initiated before the assessment were excluded. Furthermore, 28 patients with missing data on the main variables were excluded. These 28 patients included 10 who were discharged before assessment, nine with disease aggravation, three with severe behavioral and psychological symptoms of dementia, two with low arousal, and four others. Finally, 590 participants (187 males and 403 females) were included in this study (Fig. 1).

### Time points of data collection

Informed consent was obtained immediately after the transfer from an acute care ward to a community-based integrated care ward. All data were collected from the community-based integrated care ward. Data on pre-hospitalization conditions were collected when family members or their representatives visited the hospital because of admission procedures, meetings with patients, and consultations with physicians. Since the participants presented with cognitive decline, data were collected from family members or their representatives during pre-admission.

### Measurement of the dependent variable

#### Emotional distress

Emotional distress was assessed using the GDS-15 [38] as a measure of psychological adjustment during post-acute care. Participants were asked to answer “yes” or “no” to each of the 15 items (score range: 0-15). The GDS-15 is a scale for assessing depression in older adults and is useful for assessing depressive symptoms in older adults with MMSE scores of 10 or higher [37]. The Cronbach’s alpha in this study was 0.82.

### Measurement of independent variable

#### Physical function at post-acute care

The Japanese version of the Functional Independence Measure (FIM) 3.0 [39–41] was used to assess physical function. The FIM is an 18-item scale that assesses the ability to perform activities of daily living, including self-care, sphincter control, transfer, and locomotion. Each item was rated on a seven-point scale (7 = complete independence, 6 = modified independence, 5 = supervision, 4 = minimal assistance, 3 = moderate assistance, 2 = maximal assistance, and 1 = total assistance) through therapist observation. The 18 FIM items have a total score range of 18-126, with higher scores indicating higher physical function after acute care. The Cronbach’s alpha in this study was 0.96.

### Measurement of control variables

#### Physical function at pre-hospitalization

Physical function data at pre-hospitalization were retrospectively collected from the family members of patients during post-acute care. Eighteen activities of daily living, similar to those in the FIM criteria, were examined to assess the patient’s ability during prehospitalization. Each item was rated on a seven-point scale (7 = complete independence, 6 = modified independence, 5 = supervision, 4 = minimal assistance, 3 = moderate assistance, 2 = maximal assistance, and 1 = total assistance) by their own family. The total score for the 18 items ranged from 18 to 126, with higher scores indicating higher physical function pre-hospitalization. Cronbach’s alpha in this study was 0.95.

#### Cognitive function

The participants’ cognitive function in post-acute care was assessed using the MMSE [42]. The Cronbach’s alpha in this study was 0.87.

#### Sociodemographic characteristics

Data on age, sex (male and female), marital status (married, widowed, and others), residence at prehospitalization (private home and institution), level of daily living independence at prehospitalization (independence, supervision/minimal assistance, moderate assistance, and maximal assistance), duration of stay in the acute care unit, hospitalization-causing disease, and comorbidities (yes/no) were collected through interviews with family members and medical records. Hospitalization-causing diseases were defined as the immediate causes of acute hospitalization. Comorbidities were defined as the presence of diseases other than hospitalization-causing diseases.

### Statistical analysis

The age quartiles of the participants were 78.8, 83.0, and 88.0 years. Given the common practice of categorizing age in five-year increments, the participants were divided into four age groups (65-79, 80-84, 85-89, and ≥ 90 years). Descriptive statistics were calculated for each group. Differences among the four age groups were examined using one-way analysis of variance (ANOVA) for continuous variables and the chi-squared test for categorical variables. Associations among the main variables in the four age groups were examined using Pearson’s product–moment correlation coefficients. Hierarchical multiple regression analysis (forced entry method) was used to examine whether age had a moderating effect on the relationship between physical functioning during post-acute care and emotional distress. In Step 1, physical function at prehospitalization and cognitive function at post-acute care were considered control variables. Physical function during post-acute care was considered an independent variable in Step 2, whereas age was considered a moderating variable. The interaction term constructed with the independent and moderating variables was entered in step 3. Centering of means was applied to reduce multicollinearity [43]. For the physical function at post-acute care and age, each was subtracted from the mean and entered into the regression equation. Multicollinearity in the hierarchical multiple regression analysis was examined using a variance inflation factor (VIF), and a value of 10 or more was considered to be indicative of multicollinearity. For significant interaction terms, a simple slope analysis was conducted with age at − 1 SD and + 1 SD [43, 44].

Hierarchical multiple regression analysis was run on R version 4.1.3 (packages: “car” version 3.0-13, “carData” version 3.0-5, “pequod” version 0.0-5, “jtools” version 2.2.0, “ggplot2” version 3.3.6). All other analyses were conducted using IBM SPSS Statistics for Windows version 27. For all analyses, the significance level was set at a *p*-value lower than 0.05.

## Results

Table 1 summarizes the characteristics of the participants according to their age group. The mean age was 82.8 ± 6.8 years (range: 65-100), and the mean score for cognitive function (MMSE) was 21.9 ± 5.4. Of the participants, 48.8% were independent in daily living at pre-hospitalization, and the mean score for physical function at pre-hospitalization was found to be 105.1 ± 22.1.

**Table 1:** Characteristics of participants by age groups (in years)

Mean ± SD or n (%)	Total (*N* = 590)	65-79 (*n* = 172)	80-84 (*n* = 166)	85-89 (*n* = 160)	≥ 90 (*n* = 92)	*P-*Value	Effect size
Age	82.8 ± 6.8						
Female	403 (68.3)	115 (66.9)	101 (60.8)	114 (71.3)	73 (79.3)	^*^	0.13
Marital status						^***^	0.29
Married	252 (42.7)	104 (60.5)	84 (50.6)	48 (30.0)	16 (17.4)		
Widowed	305 (51.7)	48 (27.9)	74 (44.6)	107 (66.9)	76 (82.6)		
Others	33 (5.6)	20 (11.6)	8 (4.8)	5 (3.1)	0 (0.0)		
Residence at pre-hospitalization						0.37	0.07
Private home	569 (96.4)	166 (96.5)	162 (97.6)	151 (94.4)	90 (97.8)		
Institution	21 (3.6)	6 (3.5)	4 (2.4)	9 (5.6)	2 (2.2)		
Level of daily living independence at pre-hospitalization						^***^	0.15
Independence	288 (48.8)	86 (50.0)	103 (62.0)	72 (45.0)	27 (29.3)		
Supervision / Minimal assistance	238 (40.3)	60 (34.9)	46 (27.7)	75 (46.9)	57 (62.0)		
Moderate assistance	51 (8.6)	21 (12.2)	13 (7.8)	9 (5.6)	8 (8.7)		
Maximal assistance	13 (2.2)	5 (2.9)	4 (2.4)	4 (2.5)	0 (0.0)		
Physical function at pre-hospitalization	105.1 ± 22.1	105.0 ± 24.7	108.9 ± 22.3	105.1 ± 19.7	98.5 ± 19.0	^**^	0.02
Cognitive function (MMSE)	21.9 ± 5.4	23.8 ± 5.2	22.4 ± 5.1	21.0 ± 5.2	19.1 ± 5.3	^***^	0.09
Physical function (FIM)	83.0 ± 25.2	87.4 ± 25.7	86.5 ± 25.0	80.7 ± 23.8	72.6 ± 23.8	^***^	0.04
Emotional distress (GDS-15)	6.7 ± 3.8	6.9 ± 3.8	6.6 ± 3.9	6.7 ± 3.7	6.7 ± 3.8	0.95	0.00
≤ 4	193 (32.7)	50 (29.1)	52 (31.3)	57 (35.6)	34 (37.0)		
5**-**9	246 (41.7)	73 (42.4)	77 (46.4)	63 (39.4)	33 (35.9)		
≥ 10	151 (25.6)	49 (28.5)	37 (22.3)	40 (25.0)	25 (27.2)		
Duration of stay in the acute care unit, days	28.1 ± 19.7	29.1 ± 19.1	28.1 ± 22.2	27.0 ± 17.1	28.0 ± 20.7	0.84	0.00
Disease causing hospitalization						^**^	0.16
Vertebral fracture	193 (32.7)	41 (23.8)	56 (33.7)	61 (38.1)	35 (38.0)		
Femoral fracture	51 (8.6)	8 (4.7)	11 (6.6)	23 (14.4)	9 (9.8)		
Other fractures	40 (6.8)	18 (10.5)	9 (5.4)	8 (5.0)	5 (5.4)		
Other orthopaedic diseases	54 (9.2)	17 (9.9)	17 (10.2)	12 (7.5)	8 (8.7)		
Heart failure	48 (8.1)	12 (7.0)	10 (6.0)	13 (8.1)	13 (14.1)		
Pneumonia	33 (5.6)	11 (6.4)	8 (4.8)	10 (6.3)	4 (4.3)		
Parkinson's disease/Parkinson's disease with dementia	25 (4.2)	13 (7.6)	8 (4.8)	2 (1.3)	2 (2.2)		
Stroke	18 (3.1)	7 (4.1)	5 (3.0)	2 (1.3)	4 (4.3)		
Others	128 (21.7)	45 (26.2)	42 (25.3)	29 (18.1)	12 (13.0)		
Comorbidity, yes	578 (98.0)	169 (98.3)	160 (96.4)	157 (98.1)	92 (100.0)	0.25	0.08

One-way ANOVA was used for continuous variables, and chi-square test was used for categorical variables to explore differences among four age groups. The effect size for one-way ANOVA was calculated by eta squared, and chi-squared test was calculated by Cramer’s V

^*^p < 0.05, ^**^p < 0.01, ^***^p < 0.001

Alternatively, the mean score for physical function (FIM) at post-acute care was 83.0 ± 25.2, with a mean score of 4.6 points per item; a score indicating that the mean level of supervision was representing minimal assistance. The mean score for emotional distress (GDS-15) was 6.7 ± 3.8, with 41.7% of the participants scoring between 5 and 9, and 25.6% scoring 10 or more. The mean duration of stay in the acute care unit was 28.1 ± 19.7 days, and 98.0% of the participants had comorbidities.

Differences between the four age groups were examined in terms of post-acute care physical function and emotional distress. Continuous variables were assessed using a one-way ANOVA, and categorical variables were assessed using the chi-squared test. Results showed that the physical function at post-acute care was significantly different (*F*(3,586) = 8.90, *p* < 0.001, *η*^2^ = 0.04), and multiple comparisons using the Bonferroni method showed that the 65-79 years age group (*p* < 0.001) and the 80-84 years age group (*p* < 0.001) were higher than the ≥ 90 years age group. Emotional distress in post-acute care (*F*(3,586) = 0.12, *p* = 0.95, *η*^2^ = 0.00) was not significantly different among the four age groups.

### Correlations among the main variables among the four age groups

Table 2 presents the results of the Pearson’s product–moment correlation coefficients between the main variables among the four age groups. There was a significant negative correlation between the physical function at post-acute care and the emotional distress in the two younger age groups (65-79 and 80-84 years), but there was no significant correlation between them in the two older age groups (85-89, and ≥ 90 years).

**Table 2:** Correlations between the main measures by age groups of the study

		**1**		**2**		**3**	
65-79 years (*n* = 172)							
1	Physical function at pre-hospitalization						
2	Cognitive function (MMSE)	0.50^***^					
3	Physical function (FIM)	0.68^***^		0.64^***^			
4	Emotional distress (GDS-15)	− 0.22^**^		− 0.22^**^		− 0.36^***^	
80-84 years (*n* = 166)							
1	Physical function at pre-hospitalization						
2	Cognitive function (MMSE)	0.47^***^					
3	Physical function (FIM)	0.64^***^		0.56^***^			
4	Emotional distress (GDS-15)	− 0.20^**^		− 0.25^**^		− 0.28^***^	
85-89 years (*n* = 160)							
1	Physical function at pre-hospitalization						
2	Cognitive function (MMSE)	0.54^***^					
3	Physical function (FIM)	0.65^***^		0.65^***^			
4	Emotional distress (GDS-15)	− 0.17^*^		0.01		− 0.11	
≥ 90 years (*n* = 92)							
1	Physical function at pre-hospitalization						
2	Cognitive function (MMSE)	0.41^***^					
3	Physical function (FIM)	0.49^***^		0.46^***^			
4	Emotional distress (GDS-15)	− 0.07		0.05		− 0.12	

Symbols used: ^*^p < 0.05, ^**^p < 0.01, ^***^p < 0.001

### Moderating effect of age on the relationship between the physical function at post-acute care and the emotional distress

Hierarchical multiple regression analysis (forced entry method) was used to examine the moderating effect of age on the relationship between physical function in post-acute care and emotional distress (Table 3). Physical and cognitive functions at pre-hospitalization were entered as control variables in Step 1. Physical function at post-acute care was entered as an independent variable in Step 2, and age was entered as a moderating variable in Step 2. The results revealed a significant increase in the coefficient of determination from Steps 1 to 2. After controlling for the effects of prehospitalization physical and cognitive function, post-acute care physical function was significantly associated with emotional distress. Age was not found to be directly associated with emotional distress. Furthermore, when the independent variable was multiplied by a moderating variable (age) in Step 3 and entered as an interaction term, the increase in the coefficient of determination from Steps 2 to 3 was significant. The interaction term (physical function × age) entered in Step 3 was significant. The overall model explained 6.9% of the emotional distress (6.1% in adjusted *R*^2^). The variance inflation factors (VIFs) were all less than 3, and it was determined that no multicollinearity would affect the results.

**Table 3:** Hierarchical regression analysis of the emotional distress (GDS-15) at post-acute care

	**Step 1**				**Step 2**				**Step 3**			
	***B***	***SE(B)***	***β***		***B***	***SE(B)***	***β***		***B***	***SE(B)***	***β***	
Physical function at pre-hospitalization	− 0.03	0.01	− 0.16^***^		− 0.01	0.01	− 0.06		− 0.01	0.01	− 0.05	
Cognitive function (MMSE)	− 0.02	0.03	− 0.03		0.03	0.04	0.04		0.03	0.04	0.05	
Age _c					− 0.02	0.02	− 0.04		− 0.02	0.02	− 0.03	
Physical function (FIM) _c					− 0.03	0.01	− 0.23^***^		− 0.04	0.01	− 0.23^***^	
Physical function (FIM) _c × Age _c									0.00	0.00	0.11^**^	
*R*^*2*^* (R*^*2*^* adj.)*	0.03 (0.03)				0.06 (0.05)				0.07 (0.06)			
*ΔR*^*2*^	0.03^***^				0.02^***^				0.01^**^			

“_c” is the variable that was submitted to mean centering. Symbols used: ^**^p < 0.01, ^***^p < 0.001

**Fig. 2:**
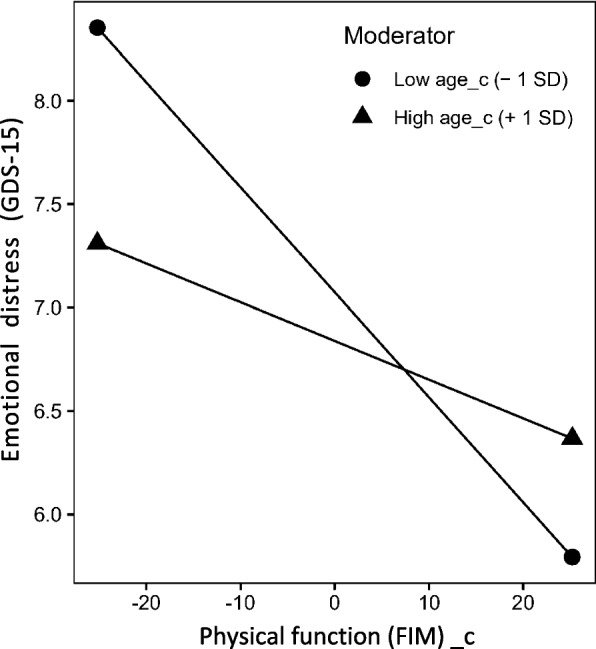
Moderating effects of aging on the relationship between physical function and emotional distress at post-acute care

A simple slope analysis was conducted because the interaction term was significant. The mean age − 1 SD and mean age + 1 SD were substituted into the regression equation [43, 44]. Figure 2 shows the results of the simple slope analysis of the physical function × age interaction term for post-acute care. When the age was the mean − 1 SD years (76.0), an association between physical function and emotional distress was observed, with the lower physical function indicating higher emotional distress (*β* =  − 0.05, t(584) =  − 4.67, *p* < 0.001). Contrastingly, no such significant association was found when the age was the mean + 1 SD years (89.6) (*β* =  − 0.02, t(584) =  − 1.76, *p* = 0.08). In other words, the relationship between physical function and emotional distress in post-acute care differs by age.

## Discussion

This study aimed to clarify whether the relationship between loss of physical health and depressed mood differs with age in older inpatients after acute care. To the best of our knowledge, few studies have examined the effects of age on the psychological adjustment of hospitalized older adults immediately after physical health loss.

The prevalence of depression in community-dwelling older adults was not higher than in other age groups [9]. However, older hospitalized patients are known to have a higher risk for depression [9, 10, 22]. In this study, 67.3% of older patients had GDS-15 scores of 5 or higher, suggesting that some older inpatients experienced emotional distress immediately after health loss.

The results of the one-way ANOVA revealed that despite poorer physical function in older patients, their emotional distress was not necessarily worse than that in younger patients. Furthermore, the results of the correlation analysis revealed that the relationship between physical function at post-acute care and emotional distress was different between the two younger age groups (65-79 and 80-84 years) and the two older age groups (85-89 and ≥ 90 years). Physical function was related to emotional distress in the two younger age groups, but no such association was found in the two older age groups. The results of this study do not provide a clear explanation of why this relationship differed dramatically after the mid-80 s. However, the data for this study were collected from 2015 to 2020, and as of 2016, the healthy life expectancy in Japan was 72.14 years for men and 74.79 years for women, while the life expectancy was 80.98 years for men and 87.14 years for women [45]. In other words, the mid-80s represent a period of transition from the third to the fourth age [29], which might have been perceived as a bonus for those in the general population who have managed to survive beyond this age window. When faced with adversity that is difficult to control, such as having serious illnesses or age-related loss, people are more likely to make social comparisons (especially downward comparisons) and try to maintain good feelings by feeling better off than others [18, 19, 46–48]. Such downward comparisons play an important role, especially in the early stages of the psychological adjustment process to serious illnesses [49] and help protect mental health under threat [48–50]. If older patients aged 85 years and above referred to their same-generation peers when facing health loss, it is possible that even if their physical function was declining, they would perceive themselves in sufficiently good health compared to their peers who had already died and would be grateful to be still alive beyond the average life expectancy of their age, rather than being discouraged by their health loss.

Previous studies have indicated that in frail and old individuals with a significantly deteriorating health status, psychological functioning is also reduced and emotion regulation is more difficult because of increased overall frailty [29–31]. However, the results of hierarchical multiple regression and simple slope analyses in this study indicated that there was a moderating effect of age, and older patients (mean + 1 SD years) showed the impact of losing physical health with weak emotional distress compared to younger patients (mean − 1 SD years). Although previous studies comparing middle-aged and older adults have suggested that age moderates the relationship between physical health loss and depression [14], we found a similar moderating effect of age in hospitalized older patients after acute treatment.

It is possible to consider that an optimistic attitude toward health may diminish motivation for rehabilitation and treatment. However, prior research reflects that even in poor health conditions, an optimistic attitude and high levels of positive emotions may lead to the active use of personal resources, which can be a predictor of good functional maintenance and life expectancy [51–53] and may be beneficial for functional recovery after hospital discharge [54]. Therefore, maintaining an optimistic attitude among older hospitalized patients after acute care without being too disappointed with their own health may have a protective effect on functional maintenance and survival. These findings would not be so inconsistent with the suggestion [32, 33] that psychological functions related to emotion regulation develop throughout life with aging [6, 8, 15–17] and are maintained even in old age when frailty is increasing.

### Limitations

In this study, emotional distress was assessed only once using the GDS-15 immediately after acute treatment, and longitudinal changes were not examined. Therefore, emotional distress did not meet the diagnostic criteria for depression and might represent a temporary change in adjustment disorders associated with acute inpatient treatment. Furthermore, some patients recovered from emotional distress with improvements in physical health. The effects of depression and physical health loss are bidirectional [2, 55, 56], and it is possible that not only does poor physical health affect depression, but depression in turn, also affects poor physical health. It has also been suggested that people with more positive emotions are more likely to survive longer [5, 33]. Given these complexities, the direction of causality could not be determined. In other words, poor physical health after acute treatment may have affected emotional distress, or emotional distress may have had a negative impact on physical recovery after acute treatment. Additionally, although individuals taking medication for depressive or bipolar disorder were excluded from this study, other patients, such as those suffering from stroke, Parkinson’s disease, or dementia, may experience increased depression due to the disease itself [2, 9, 10], and it is quite possible that these factors might have influenced the results of this study. Moreover, the participants of this study were Japanese, and cultural factors (such as the unique Japanese view of life and death, as well as Japanese religion) may have influenced our results.

## Conclusions

We found a moderating effect of age on the relationship between physical health loss and emotional distress among older inpatients receiving acute care. We found that relatively younger and older patients differ in their psychological vulnerability when faced with physical health loss following acute treatment, and for similar levels of physical health loss, older patients were less likely to experience emotional distress. The cause of this phenomenon remains unclear, but a common background mechanism may be at work to maintain higher levels of mental health in the oldest old.

## Data Availability

The datasets generated and analyzed in this study are not available to the public because of participant confidentiality but are available from the corresponding author upon reasonable request.

## Abbreviations

ANOVA: Analysis of variance
FIM: Functional independence measure
GDS: Geriatric depression scale
MMSE: Mini-mental state examination
SD: Standard deviation
SIAS: Stroke impairment assessment set
SOEP: Socio-economic panel
VIF: Variance inflation factor
